# Editorial: Investigating drugs used off-label in various cancers

**DOI:** 10.3389/fonc.2023.1130834

**Published:** 2023-01-18

**Authors:** Wang Liu, Benyi Li

**Affiliations:** Department of Urology, University of Kansas Medical Center, Kansas City, KS, United States

**Keywords:** cancer treatment, drug reposition, drug repurposing, human cancers, anti-cancer medication

Cancer drug discovery is a long, expensive, drawn-out process involving the identification and optimization of lead compounds, followed by pre-clinical testing and clinical trials (1). It has been proved that its span varies between 11.4 to 13.5 years, and the costs range from 161 to 1800 million dollars from initial experiments to completed regulatory reviews (2, 3). Currently, more than 10,000 clinical trials investigating drug candidates in cancer are registered at www.clinicaltrials.gov. However, only a limited number of drug candidates progress to the next phase in clinical trials, with reports showing that less than 5% of drug compounds can be approved to enter phase I trials (4). Collectively, there is an urgent need to expand more discovery methods to remedy the growing anti-tumor medication needs gap.

Drug repurposing or repositioning is the application of a drug for another indication than its original application (5). Discovering that already licensed medication readily available in the market affects diseases otherwise not knowingly attributed to these drug molecules avoids this research process. It can bring new, potentially life-saving treatments to patients with a cheaper cost and a faster path. However, repositioning the drug for further indication may accompany side effects not previously reported and will require validation in a new clinical trial. Nevertheless, the safety profile can be referred to as the initial indication, thus, increasing the likelihood of the drug through the trial (1).

Recent clinic reports show that several epidemiological studies reveal lower cancer incidence in individuals receiving long-term psychotropic drug treatment. Varalda et al. investigated psychotropic drugs for their anti-tumor activity and evaluated their cytotoxic activity in colorectal carcinoma, glioblastoma, and breast cancer cell lines. The investigation revealed that penfluridol, ebastine, pimozide, fluoxetine, fluspirilene, and nefazodone have apparent cytotoxicity in all cancer cell lines tested in the low micromoles range. These psychotropic drugs caused mitochondrial membrane depolarization, increased the acidic vesicular compartments, and induced phospholipidosis in breast cancer MCF-7 cells. Varalda et al. showed that psychotropic drugs *via* dual targeting of lysosomes and mitochondria are a novel promising approach to cancer therapy, especially for cancer cells deficient in apoptotic machinery. Chlorpromazine has been used to treat psychiatric disorders for more than six decades. Clinic reports show that chlorpromazine is a potential anti-tumor medicine, but the mechanism is unclear. Matteoni et al. tested the anti-tumor effect of chlorpromazine in six glioblastoma cell lines. The results showed that chlorpromazine inhibited cancer cell viability in an apoptosis-independent way, induced hyperdiploidy, reduced cloning efficiency, neurosphere formation, and downregulated the expression of stemness genes. Furthermore, combining chlorpromazine with temozolomide, the first-line therapeutic in GBM patients, significantly inhibited cell growth and cloning efficiency in GBM cell lines.

Volatile anesthetics, such as inhalation anesthetics in clinical anesthesia, were found to regulate cancer-related signaling. Wang et al. systematically summarize the research progress of volatile anesthetics in anti-cancer signaling regulation. It provided good insights for guiding clinical anesthesia procedures and instructing to enhance recovery after surgery. Cyclovirobuxine D (CVBD) is a triterpenoid alkaloid extracted from *Buxus Sinica* and other plants of the same genus used in cardiomyopathy, myocardial infarction, and arrhythmia (6, 7). Accumulating evidence showed that CVBD has a potential anti-tumor effect in multiple tumor cell types. Zhang et al. investigated the anti-cancer effect of CVBD on GBM and showed that CVBD has a significant anti-proliferation effect on the T98G and U251 cell lines. CVBD also induced apoptosis and mitochondrial damage in GBM cells. Mechanistically, CVBD caused cofilin mitochondrial translocation and superoxide species accumulation in mitochondria in a dose-dependent manner. In addition, Li et al. found that both anti-helminthic and anti-protozoal drugs suppressed tumor growth by targeting multiple pathways *via* different mechanisms. They suggested further evaluation of these anti-parasitic drugs for cancer therapy.

Magnesium (Mg^2+^), the second most predominant intracellular cation, plays an essential role in many physiological functions. Several reports showed that the high intracellular concentration of magnesium contributes to cancer initiation and progression in various cancers. To investigate the effect of magnesium concentration in cancer therapy, Li et al. designed a series of experiments to assess the underlying mechanisms in bladder cancer both *in vitro* and *in vivo*. The results indicated that cancer cell proliferation was inhibited in a high concentration of MgCl_2_ or MgSO_4_ treatment. MgCl_2_ treatment induces apoptosis, G_0_/G_1_ cell cycle arrest, autophagy, and ER stress but not cell migration. Combinational treatment of MgCl_2_ and VPA dramatically reduced the proliferation, migration, and *in vivo* tumorigenicity. Vitamin D is a lipid-soluble hormone that promotes skeletal mineralization and maintains calcium homeostasis by binding to the vitamin D receptor. Vitamin D also involves cell proliferation, angiogenesis, apoptosis, inflammation, and cell difference. Adelani et al. had a review article summarizing the functions of vitamin D in Hepatocellular carcinoma (HCC). They discussed the specific therapeutic targets from *in vivo*, *in vitro*, and clinical studies. They elucidated that vitamin D-associated target genes have essential functions in the anti-tumor effect through inflammation, oxidative stress, invasion, and apoptosis pathways.

Combining different chemotherapy medications is also an excellent method to improve the anti-tumor effect and overcomes chemotherapy resistance. Atorvastatin is a popular preventive medicine for cardiovascular diseases. Yuan et al. used Atorvastatin plus dexamethasone in two patients with leukemia-related chronic subdural hematoma. However, this combinational therapy was not effective for a patient with leukemia-related encephalopathy. Fan et al. used Ascorbate plus tyrosine kinase inhibitors (TKIs) in hepatocellular carcinoma cells. They found that Ascorbate enhanced TKI’s efficacy in HCC cells by disturbing redox homeostasis. Yang et al. screened the Human Epigenetic Drug Database and tested the positive lead compounds in cytotoxic experiments. Their results showed that the anti-hypertension drug Hydralazine reduced the overall incidence rate in most subgroups of hematologic neoplasms when chronically used at a low dose. Song et al. presented a complete analysis of the historical progression in Temozolomide (TMZ) development and suggested several research directions for future research.

In conclusion, this Research Topic had a thriving collection of 11 articles on repurposing multiple existing medications for anti-cancer treatment. These research results significantly contributed to the development of novel cancer therapies.

## Author contributions

WL drafted the manuscript. BL revised and approved the submission.

## References

[1] Sleire L Førde HE Netland IA Leiss L Skeie BS Enger PØChecktae. Drug repurposing in cancer. Pharmacol Res (2017) 124:74–91. 28712971 10.1016/j.phrs.2017.07.013

[2] Paul SM Mytelka DS Dunwiddie CT Persinger CC Munos BH Lindborg SR. How to improve R&D productivity: the pharmaceutical industry's grand challenge. Nat Rev Drug Discovery (2010) 9(3):203–14. 10.1038/nrd3078 20168317

[3] Adams CP Brantner VV. Estimating the cost of new drug development: is it really 802 million dollars? Health Aff (Millwood) (2006) 25(2):420–8. 10.1377/hlthaff.25.2.420 16522582

[4] Hay M Thomas DW Craighead JL Economides C Rosenthal J. Clinical development success rates for investigational drugs. Nat Biotechnol (2014) 32(1):40–51. 24406927 10.1038/nbt.2786

[5] Ashburn TT Thor KB. Drug repositioning: identifying and developing new uses for existing drugs. Nat Rev Drug Discovery (2004) 3(8):673–83. 10.1038/nrd1468 15286734

[6] Guo Q Guo J Yang R Peng H Zhao J Li L. Cyclovirobuxine d attenuates doxorubicin-induced cardiomyopathy by suppression of oxidative damage and mitochondrial biogenesis impairment. Oxid Med Cell Longev 2015:151972. 26075032 10.1155/2015/151972 PMC4446494

[7] Yu B Fang T-H Lü G-H Xu H-Q Lu J-F. Beneficial effect of cyclovirobuxine d on heart failure rats following myocardial infarction. Fitoterapia (2011) 82(6):868–77. 10.1016/j.fitote.2011.04.016 21575690

